# Comparison of functional outcomes in patients with lumbar canal stenosis with and without obesity treated with lumbar decompression surgery

**DOI:** 10.3389/fsurg.2026.1731137

**Published:** 2026-02-12

**Authors:** Ke Zhou, Bin Yu, Kaifeng Gan, Dikai Bei, Binhui Chen, Jie Li

**Affiliations:** Department of Orthopaedic Surgery, The Affiliated Lihuili Hospital of Ningbo University, Ningbo, Zhejiang, China

**Keywords:** BMI, clinical outcomes, linear regression analysis, lumbar decompression surgery, obesity

## Abstract

**Objective:**

The rising prevalence of obesity has raised concerns about its impact on surgical outcomes. Obesity is a critical risk factor of low back pain and lumbar degeneration diseases, but it is still unclear whether obesity is related to lumbar surgical outcomes in the Chinese population. This study examines the influence of body mass index (BMI) on the clinical effectiveness of lumbar decompression surgery in patients with lumbar canal stenosis (LCS).

**Methods:**

465 patients with single-segment LCS treated with lumbar decompression surgery at the LiHuiLi Hospital between April 2018 and August 2023 were enrolled in this study. Patients were divided into obesity (OB, BMI > 30 kg/m²) and non-obesity (NOB, BMI < 24 kg/m²) groups. Baseline data, operation time (OT), hospital stay (HS), Visual Analogue Scale (VAS) scores, Oswestry Disability Index (ODI), and maximum walking distance (MWD), C-reaction protein (CRP), white blood cell (WBC), complications, and reoperations were evaluated.

**Results:**

Of the total participants, 156 were categorized as OB and 309 as NOB. The NOB group exhibited significantly greater improvement in VAS scores at 1 month and ODI scores at 3 and 12 months postoperatively (*P* < 0.001). The OB group had significantly lower MWD both preoperatively and at the final follow-up compared to the NOB group (*P* < 0.001). The OB group also had significantly longer OT and HS (*P* < 0.001), as well as higher rates of complications and reoperations compared to the NOB group (*P* < 0.05). Linear regression revealed a significant relationship between BMI and MWD (*P* < 0.001).

**Conclusions:**

Obesity maybe associated with poorer functional recovery, increased complications, and prolonged recovery following lumbar decompression surgery.

## Introduction

Lumbar canal stenosis (LCS) is a common condition that results in leg and/or back pain and reduced function, particularly in the elderly population. It is often associated with symptoms such as claudication or leg pain (1). LCS is typically caused by disc degeneration, ligamentum flavum hypertrophy, and facet joint hypertrophy. Initial management usually involves conservative treatments such as analgesics or physiotherapy. However, if symptoms persist despite conservative methods, surgical intervention may be required. The primary objective of surgery is to achieve complete decompression of the spinal canal (including the ligamentum flavum, fat, and osteophytes) and to ensure lumbar stability (2), thereby improving walking ability and increasing overall physical activity.

Obesity has been identified as an independent predictor of back pain and is often associated with lumbar disc herniation and LCS (3, 4). Patients with LCS often experience lower limb claudication, which limits walking distance and reduces physical activity. This decline in activity can further increase body mass index (BMI), exacerbating lumbar degeneration and canal stenosis. Additionally, obesity increases the risk of cardiovascular diseases, diabetes, and osteoarthritis, all of which contribute to a decline in quality of life (5, 6). With the rising prevalence of obesity, an increasing number of patients with LCS are also obese. The World Health Organization defines obesity as a BMI greater than 30 kg/m², and it is believed to negatively impact the treatment of LCS. As a result, numerous studies have investigated the efficacy of lumbar decompression surgery in obese patients, focusing on its effects on clinical outcomes, complications, and maximum walking distance.

Recent studies have reported that obesity may negatively impact outcomes in patients undergoing lumbar surgery. Bergquist et al. examined 174 patients with LCS who underwent full-endoscopic unilateral laminotomies for bilateral decompression, of whom 74 were obese and 100 had normal weight. Their findings indicated that while endoscopic techniques can partially mitigate obesity-related surgical morbidity, obesity was significantly associated with increased postoperative analgesic use (7). Similarly, Hareni et al. analyzed preoperative and 1-year postoperative data from 14,984 patients in the National Swedish Quality Registry for Spine Surgery and found that morbidly obese patients had more complications than those with lower BMI (*P* < 0.05) (8). Krüger et al. reported higher complication rates, greater postoperative analgesic requirements, and significantly lower reductions in lower back pain among obese patients (BMI > 40 kg/m²) (9).

The impact of obesity on postoperative complications following lumbar decompression surgery remains a topic of debate. To our knowledge, few studies explored the relationship between obesity and clinical outcomes in Chinese patients with LCS. Therefore, this study aims to investigate the influence of obesity (BMI > 30 kg/m²) on preoperative functional outcomes and to analyze the correlation between BMI and postoperative outcomes. We hypothesize that obesity is associated with poorer recovery and increased complications.

## Methods

A total of 465 eligible patients who underwent lumbar decompression surgery at the Affiliated LiHuiLi Hospital of Ningbo University between April 2018 and September 2023 were enrolled in this study. Baseline characteristics and functional outcomes were obtained from medical records. The study was approved by the Ethics Committee of the hospital (Approval No.: LHL-2025-045).

All cases were diagnosed based on symptoms, physical examination, and radiological findings (MRI), and failure to respond to at least 3 months of conservative treatment. The inclusion criteria were: (1) age between 25 and 70 years; (2) BMI > 30 kg/m² or < 24 kg/m²; (3) back pain with leg pain or intermittent claudication; (4) single-segment LCS treated with lumbar decompression surgery; and (5) a minimum of one year of follow-up with complete data. Exclusion criteria included: (1) previous lumbar surgery; (2) spinal conditions such as fractures, scoliosis, or tumors that could interfere with postoperative outcomes; (3) systemic diseases, including rheumatoid arthritis, orthopedic tuberculosis, osteoporosis, chronic renal failure, or cardiac failure, that could influence preoperative scores or postoperative recovery.

**Figure 1:**
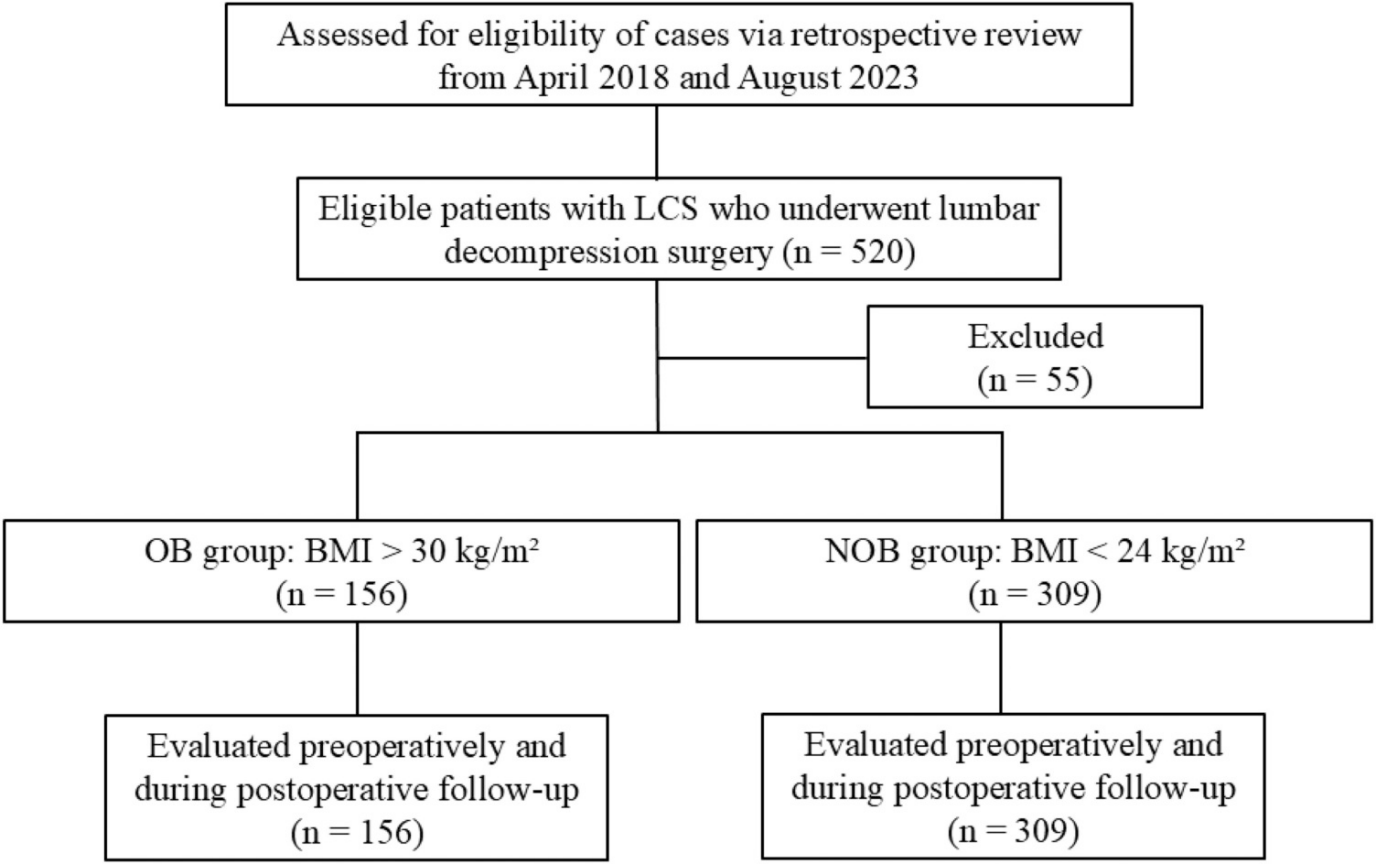
Study flowchart following the consolidated standards of reporting trials (CONSORT) guidelines. LCS, lumbar canal stenosis; OB, obesity; NOB, non-obesity; BMI, body mass index.

Initially, 520 patients with LCS were enrolled in this study. After applying the exclusion criteria, 55 cases were excluded, resulting in a final cohort of 465 eligible patients. These patients were categorized into two groups based on BMI: the obesity (OB) group (*N* = 156) and a non-obesity (NOB) group (*N* = 309) (Figure 1).

### Surgical procedure of lumbar decompression surgery

Each patient underwent tracheal intubation under general anesthesia. After installing the appropriate quadrant expandable channel, the soft tissue over the lamina and facet surface was exposed, and the segment was confirmed using a C-arm. Once the microscope was successfully positioned, the unilateral lamina (as determined by the senior surgeon), the inferior and partial superior articular process, the ligamentum flavum, and the intervertebral disc were carefully removed. This procedure decompressed the unilateral spinal canal and exposed the nerve root. A suitable-sized interbody fusion cage was then placed, followed by the insertion of pedicle screws and a connecting rod on the decompression side. The operating table was tilted to the opposite side, and after adjusting the microscope and installing the channel, the base of the spinous process and the contralateral portion of the lamina were removed. A laminar rongeur was used to dissect the ligamentum flavum until the contralateral nerve root was exposed. Pedicle screws and a connecting rod were then inserted on the opposite side. Finally, wound drainage was applied, and the incision was closed in layers.

### Postoperative rehabilitation

Postoperatively, all patients received neurotrophic drugs, muscle relaxants, and painkillers based on their symptoms. Patients were instructed to use a hard bed and wear a waist brace during daily activities for 3 months. They were also advised to avoid prolonged standing or strenuous physical labor. At the three-month follow-up visit, they were evaluated in the outpatient department, where their maximum walking distance was recorded, and they were allowed to remove the waist brace based on their tolerance. A similar assessment was conducted at the one-year follow-up visit.

### Outcome evaluation

Baseline data, including age, gender, BMI, smoking status, diabetes, operation segment, operation time, and hospital stay, were collected from medical records. Pain was assessed using the Visual Analogue Scale (VAS), which was recorded preoperatively, immediately postoperatively, and at 1-month and 1-year follow-up visits. Lumbar function was evaluated using the Oswestry Disability Index (ODI) and maximum walking distance (MWD), both recorded at the same time points (10). Additionally, inflammatory markers, including C-reactive protein (CRP) and white blood cell (WBC) count, were analyzed.

Complications were identified from medical records. Major complications, defined as those that adversely affected recovery or required intervention, included incision suppuration, cerebrospinal fluid leakage, intraspinal hematoma, internal fixation failure, and delayed bone union. Other complications, including incision redness, swelling, and pain, were classified as minor. All major second surgical procedures for the operated segment during unplanned returns to the inpatient department were defined as reoperations.

### Statistical analysis

All data were analyzed using SPSS 20.0 (Chicago, IL, USA) and presented as percentages or means ± standard deviations. Categorical variables, including gender, smoking, diabetes, complications, and reoperation rates were analyzed using Fisher's exact tests or Chi-square tests, as appropriate. Measurement data, including age, BMI, operation time (OT), length of hospital stay (LOS), VAS, ODI, and MWD, were compared using dependent *t*-tests. Linear logistic regression was used to evaluate the relationship between obesity and clinical outcomes, with results presented as odds ratios (OR) and 95% confidence intervals (CI). Kaplan–Meier survival curves were used to illustrate the relationship between complications, reoperations, and time in both groups. A *P*-value < 0.05 was considered statistically significant.

## Results

This study enrolled 465 patients with single-level LCS who underwent lumbar decompression surgery. Of these, 156 patients were categorized into the OB group (BMI > 30 kg/m²), whereas the remaining 309 patients were placed in the NOB group (BMI range: 18–24 kg/m²). Baseline characteristics for both groups are presented in Table 1. No significant differences were observed in gender, smoking status, diabetic state, or operative segment level. Additionally, preoperative VAS and ODI scores were comparable between both groups (*P* > 0.05). However, the NOB group had significantly shorter operation times (137.7 ± 10.6 min vs. 165.3 ± 8.5 min, *P* < 0.05) and hospital stays (7.9 ± 1.4 days vs. 6.6 ± 1.1 days, *P* < 0.05) compared to the OB group.

**Table 1:** Comparison of baseline characteristics between the two groups.

Characteristic	OB group	NOB group	*P* Value
Cases	156	309	
BMI (kg/m^2^)	31.7 ± 1.3	21.3 ± 1.4	<0.001*
Gender			0.54
Male	63	126	
Female	106	212	
Age (yr)	48.5 ± 11.1	48.9 ± 10.3	0.7
Smoking			0.34
Yes	70	148	
No	99	190	
Diabetic			0.06
Yes	94	161	
No	75	177	
Operation time (min)	86.8 ± 13.4	72.8 ± 12.6	<.001*
Long of hospital stay (day)	3.4 ± 1.5	2.5 ± 1.2	<.001*
Operation level			0.85
L2/3	14	31	
L3/4	19	35	
L4/5	65	119	
L5/S1	71	153	

BMI, body mass index; OB, obesity; NOB, non-obesity.

* *P* < 0.05.

**Figure 2:**
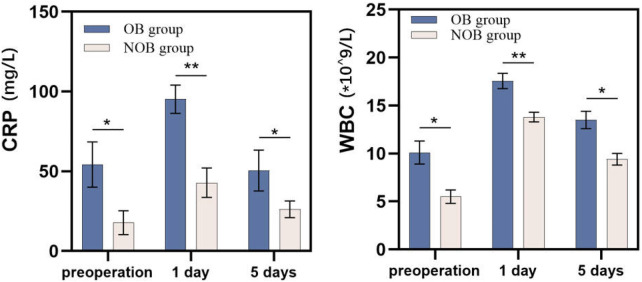
Comparison of inflammation marker levels between the two groups. CRP, C-reaction protein; WBC, white blood cell; OB, obesity; NOB, non-obesity.

The comparison of functional clinical outcomes between the two groups is presented in Table 2. At the 1-month follow-up, the NOB group showed significantly greater improvement in VAS scores compared to the OB group (1.2 ± 0.4 vs. 1.8 ± 1.7, *P* < 0.05), though no significant differences were observed at later follow-ups. Regarding ODI, the NOB group demonstrated significantly better recovery at the 3-month (16.5 ± 1.8 vs. 18.3 ± 2.9, *P* < 0.05) and 1-year (13.6 ± 1.7 vs. 15.5 ± 2.8, *P* < 0.05) follow-ups. Additionally, the OB group exhibited significantly lower maximum walking distance (MWD) at all follow-up time points (*P* < 0.05). As shown in Figure 2, the NOB group also had significantly lower levels of CRP and WBC compared to the OB group at each follow-up visit (*P* < 0.05).

**Table 2:** Comparison of clinical outcomes between the two groups.

Variables	OB group (*N* = 156)	NOB group (*N* = 309)	*P* Value
VAS			
Preoperative	5.9 ± 0.9	6.0 ± 0.7	0.24
Postoperative	2.5 ± 0.6	2.5 ± 0.8	0.23
1 month	1.8 ± 1.7	1.2 ± 0.4	<.001*
12 months	0.9 ± 0.5	1.0 ± 0.4	0.15
ODI			
Preoperative	57.9 ± 9.8	58.4 ± 10.5	0.66
3 months	18.3 ± 2.9	16.5 ± 1.8	<.001*
12 months	15.5 ± 2.8	13.6 ± 1.7	<.001*
MWD, m			
Preoperative	916.7 ± 375.7	1,257.9 ± 165.9	<.001*
3 months	1,495.5 ± 307.4	2,026.2 ± 311.4	<.001*
12 months	2,210.3 ± 456.5	2,751.8 ± 478.5	<.001*
Complications	15	11	<.001*
Reoperation	9	3	<.001*

VAS, visual analogue scale; ODI, Oswestry disability index; MWD, maximum walking distance; OB, obesity; NOB, non-obesity.

* *P* < 0.05.

**Figure 3:**
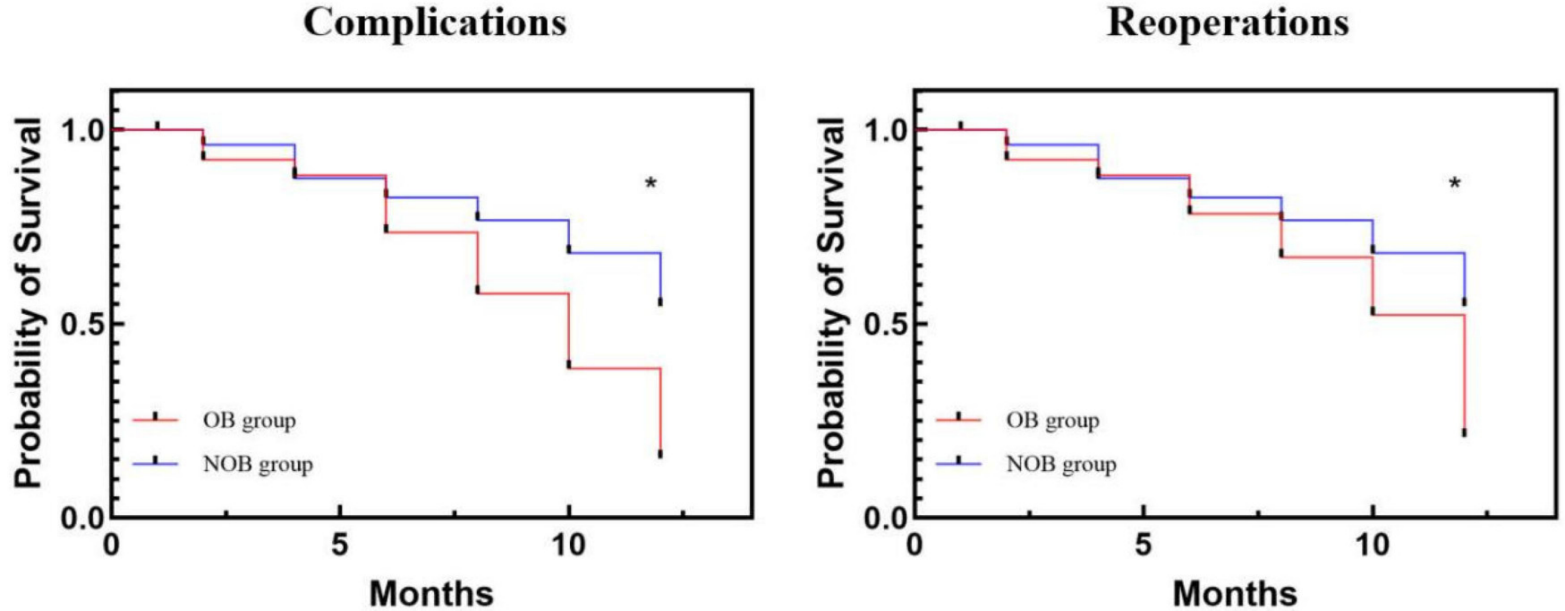
Kaplan–Meier survival curve illustrating the relationship between BMI and complications and reoperations. OB, obesity; NOB, non-obesity.

Linear logistic regression was used to analyze the relationship between BMI and clinical outcomes (Table 3). BMI showed a significant correlation with ODI at 3 months (*P* < 0.05, 95% CI = 0.314–0.573) and with VAS at the 1-month follow-up (*P* < 0.05, 95% CI = 0.175–0.752). However, BMI did not show significant correlation with VAS or ODI at other follow-up time points. For MWD, BMI was significantly associated with outcomes at each follow-up time point. The OB group had significantly more complications (15 vs. 11) and reoperations (9 vs. 3) compared to the NOB group during follow-up (*P* < 0.05). Survival analysis confirmed a significant association between obesity and complications/reoperations (Kaplan–Meier, *P* < 0.05, Figure 3).

**Table 3:** Linear regression analysis of the relationship between BMI and clinical outcomes.

Dependent variable	B	95% Confidence interval	*P* Value
VAS			
Preoperative	0.031	−0.368–0.431	0.877
Postoperative	−0.158	−0.573–0.258	0.457
1 month	0.463	0.175–0.752	<.05
12 months	0.103	−0.610–0.816	0.776
ODI			
Preoperative	−0.002	−0.032–0.027	0.87
3 months	0.443	0.314–0.573	<.05
12 months	−0.008	−0.149–0.132	0.905
MWD			
Preoperative	−0.005	−0.006 to −0.004	<.001*
3 months	−0.006	−0.006 to −0.004	<.001*
12 months	−0.002	−0.003 to −0.002	<.001*

VAS, visual analogue scale; ODI, Oswestry disability index; MWD, maximum walking distance.

* *P* < 0.05.

In the OB group, 8 patients experienced wound inflammation, 7 had back pain, 2 developed wound infections, and 1 patient developed pneumonia immediately after surgery. All these complications were successfully managed conservatively. Additionally, 9 patients experienced hyperesthesia on the lateral part of the operated leg, with symptoms gradually resolving after nerve block injections. One patient fell after standing up without a brace and was diagnosed with internal fixation failure via CT scan, necessitating reoperation. Fourteen patients experienced recurrent leg pain and neurological symptoms (positive Lasegue's sign and muscle weakness) on the operated side. Ten of these patients underwent unilateral biportal endoscopic (UBE) decompression. All reoperated patients achieved symptom relief and uneventful recovery during follow-up. In the NOB group, 11 patients had wound inflammation, 8 experienced back pain, and 1 had a wound infection. Additionally, 5 patients had hyperesthesia, and 6 had wound infections. All patients in the NOB group recovered completely with wound dressing, painkillers, and neurotrophic drugs. Three patients underwent reoperation due to recurrent neurological symptoms.

## Discussion

### Pathophysiology of lumbar canal stenosis

Epstein et al. provided the radiological description of LCS in 1977 (11). Stenosis refers to any type of narrowing of the spinal canal, nerve root canal, or intervertebral foramina, which can be caused by bone or soft tissue changes. The narrowing can affect the bony canal, the dural sac, or both. Multiple factors contribute to the development of spinal stenosis, often acting synergistically to exacerbate the condition. Degeneration of the intervertebral disc often causes protrusion, leading to ventral narrowing of the spinal canal. Additionally, the height of the intervertebral space decreases, further narrowing the recess and intervertebral foramina, and increasing the strain on the facet joints. Increased load can lead to facet joint arthrosis, hypertrophy of the joint capsules, and the development of expanding joint cysts, resulting in spinal instability (12). Moreover, the decrease in intervertebral height also contributes to hypertrophy of the ligamentum flavum.

LCS can lead to the compression of nerve roots, meninges, intraspinal vessels, and, in rare cases, the cauda equina. During physical activity, reduced arterial blood flow can cause ischemia, whereas venous congestion and nerve compression can lead to secondary perfusion deficiency, resulting in claudication (13, 14).

### Relationship between obesity and lumbar canal stenosis

Approximately 24–89 million females and 39–125 million males worldwide were obese in 2016 (15). Nishida et al. reported that obesity is a global health concern that promotes chronic low-grade inflammation, leading to insulin resistance (16). Sakai et al. analyzed the characteristics of 1,119 patients aged over 65 years who were treated for LCS and found that insulin resistance is a significant risk factor for ligamentum flavum hypertrophy (17). In contrast, Rigsby et al. collected baseline and six-month abdominal MRIs from 98 overweight or obese but otherwise healthy subjects, examining the relationship between dorsal epidural fat volumes and BMI. They found that a one-point increase in BMI corresponded to a 45 mm³ increase in dorsal epidural fat volume (*P* < 0.001, 95% CI: 31.87–76.77) (18). Therefore, obese patients are more likely to develop LCS. Besides, obese increases mechanical load, which accelerates lumbar degeneration. It is reported that lumbar IVD deformation following treadmill walking increases with increasing BMI (19). This persistent mechanical damage accelerates the loss of water in the nucleus pulposus and the decrease in the elasticity of the annulus fibrosus, leading to disc bulging or protrusion, which in turn directly occupies the spinal canal space.

### Effect of obesity on functional clinical outcomes

In this study, we collected patient data and found that the NOB group achieved significantly better improvement in VAS scores (1.2 ± 0.4 vs. 1.8 ± 1.7, *P* < 0.05) at the 1-month follow-up compared to the OB group. Additionally, significantly better recovery in ODI scores was observed in the NOB group at the 3-month (16.5 ± 1.8 vs. 18.3 ± 2.9, *P* < 0.05) and 1-year (13.6 ± 1.7 vs. 15.5 ± 2.8, *P* < 0.05) follow-ups compared to the OB group. Regarding MWD, the OB group showed significantly lower MWD at each time point compared to the NOB group (*P* < 0.05). Onyekwelu et al. compared the clinical outcomes of 2,447 patients treated with lumbar spinal surgery, of whom 1,266 were obese and 1,181 were non-obese. They found that the non-obese patients had significantly better recovery in ODI scores and less blood loss (*P* < 0.05) (20). Moreover, Kaplan–Meier survival curves for BMI, complications, and reoperations were analyzed, with the NOB group exhibiting significantly better outcomes compared to the OB group (*P* < 0.05). Similarly, Divi et al. reported contrasting outcomes in a study involving 366 patients treated with lumbar fusion surgery, who were classified into four groups based on BMI. They found no significant differences among the groups, although patients with the highest BMI tended to experience more complications (21).

Linear logistic regression analysis revealed a moderate relationship between BMI and VAS/ODI scores. However, BMI emerged as a risk factor for unfavorable MWD outcomes at each time point (*P* < 0.05). Possible explanations for this include: (1) Obesity leads to metabolic dysfunction and increased biomechanical load, which may promote fat infiltration in the paravertebral muscles (22). This excess load on the spine, combined with compromised lumbar support, could account for the decreased maximum walking distance in obese patients; (2) Obese patients are more prone to metabolic dysfunction than normal-weight patients (23). Regarding CRP and WBC levels, obese patients showed significantly higher levels of inflammatory markers preoperatively compared to non-obese individuals (*P* < 0.05). This trend persisted at both 1-day and 5-day follow-ups (*P* < 0.05). He et al. reported that obese patients often exhibit metabolic dysfunction, which includes elevated adipokines and hyperglycemia. These factors may contribute to increased oxidative stress, which in turn activates inflammatory and coagulation pathways, making obese individuals more susceptible to perioperative complication (24). Obesity is not a limiting factor in surgical consideration, although obesity is associated with poorer surgery outcome. It was reported that obese population achieve considerable reduction in weight following decompression surgery for LCS, which further results in reduction in the risk of metabolic diseases (25). Besides, percutaneous endoscopic lumbar discectomy (PELD) maybe a better surgical approach for obese adolescents. A study found that PELD achieves similar outcomes in obese and normal adolescent patient with lumbar disc herniation (26). It is recommended that the patients with obesity undergo a longer rehabilitation training period and strengthen weight management after lumbar surgery.

In conclusion, obese patients may experience unfavorable functional outcomes after lumbar decompression surgery compared to those with a normal weight. The presence of excess body fat and an elevated proinflammatory state may lead to a higher incidence of perioperative complications in obese individuals. Additionally, the increased mechanical stress on the spine and insufficient lumbar support could contribute to a reduced walking capacity. Clinically, it is crucial to provide extra care for obese patients, focusing on effective wound management and encouraging weight loss as part of their overall treatment plan.

## Limitations

This study has several limitations. First, it is a retrospective, single-center study with a relatively small sample size. A large-scale, multicenter study is needed to better understand the relationship between obesity and clinical outcomes after lumbar decompression surgery. Second, radiological outcomes were not included in this study. However, clinical outcomes and walking distance are equally important indicators of surgical success.

## Data Availability

The original contributions presented in the study are included in the article/Supplementary Material, further inquiries can be directed to the corresponding author/s.
